# Effect of nanohypericum (*Hypericum perforatum* gold nanoparticles) treatment on restraint stressinduced behavioral and biochemical alteration in male albino mice

**DOI:** 10.4103/0974-8490.75450

**Published:** 2010

**Authors:** D. Jaya Prakash, S. ArulKumar, M. Sabesan

**Affiliations:** *Department of Zoology, Faculty of Science, Annamalai University, Annamalai Nagar, Tamilnadu, India*

**Keywords:** *Hypericum perforatum*, Nanohypericum, goldnanoparticles, T-maze, mirror chamber, stress

## Abstract

**Backgorund:**

*Hypericum perforatum* extract (HPE), is known for its antidepressant effect.

**Methods:**

In the present study we investigated the effect of *H. perforatum* gold nanoparticles (Nanohypericum-HPGNPs) protective role against restraint stress-induced behavioral and biochemical alterations in mice. Animals were immobilized for a period of 6 hrs/day. HPE (200 mg/kg) and nanohypericum (20 mg/kg) were administered 30 minutes before the animals were subjected to acute immobilized stress. Behavioral test parameters for anxiety and spatial memory were assessed followed by biochemical parmeters (lipid peroxidation, super oxide dismutase, catalase, glutathione peroxidase, reduced glutathione, etc.) subsequently.

**Results:**

The behavior study showed severe anxiety and memory loss compared to unstressed animals. Biochemical analyses revealed an increase in lipid per oxidation, depletion of super oxide dismutase, reduced glutathione, catalase activity and glutathione per oxidase as compared to unstressed animal. Twenty one days of *H. perforatum* and nanohypericum treatment in a dose of 200 mg/kg and 20 mg/kg, respectively, significantly attenuated restraint stress-induced behavioral and oxidative damage.

**Conclusion:**

In conclusion nanohypericum prove the modest activity than the HPE.

## INTRODUCTION

*Hypericum perforatum* L. (HP) is a medicinal plant used all over the world. The efficacy of the extract has been supported by some pharmacological and clinical studies.[1,2] attracting the interest of pharmaceutical industries. Presently, HP is one of the leading medicinal herbs sold both in Europe and in the USA.[3] HP is an increasingly popular alternative to conventional medications used for the treatment of mild to moderate depression.[4] Aggressive analysis of this plant over the last three decades has revealed that it possesses several biological properties, including antidepressant, antiviral and antiproliferative activities.[5,6] *H. perforatum* extract (HPE) has been found to contain several classes of compounds common to most plants, including flavanoids, polyphenolics, porphyrins and essential oils.[7]

Stress is a common experience of daily life and all organisms have evolved mechanisms and strategies to deal with crucial alterations in their internal and external environment. The potency to cope with or adapt to stressors is a fundamental requirement for survival. In vertebrates, diverse stressors activate a wide spectrum of interacting hormonal and neuronal systems resulting in behavioral and physiological responses. These stress responses are variable and there are individual differences both physiologically and behaviorally in how an organism perceives a perturbation and in the resulting adaptational/mal adaptational processes.[8,9] Stress induces changes in emotional behavior, anxiety-like state[10] that are associated with oxidative damage i.e., free radical damage[11,12] Acute restraint stress stimulates numerous cellular cascade that lead to increase ROS production.[13] The stress triggers the motor alteration in different animal models.[14]

Nanotechnology has recently gained attention as one of the critical research endeavors of the 21^st^ century. According to the National Nanotechnology Initiative, nanotechnology is the research and development of nanosystems, such as nanoparticles, on the scale of 1–100 nm.[15] Gold nanoparticles have found use in diagnostic and drug delivery applications.[16] Therefore, there is a growing need to develop eco-friendly nanoparticle synthesis without using toxic chemicals. Biological methods for nanoparticle synthesis using microorganisms, enzymes and plants or plant extracts have been suggested as possible eco-friendly alternatives to chemical and physical methods.[17] Using plants for nanoparticle synthesis can be advantageous over other biological processes because it eliminates the elaborate process of maintaining cell cultures and can also be suitably scaled up for large-scale nanoparticle synthesis.[18] So, the present study has been designed to evaluate the effects of *H. perforatum* gold nanoparticles (HPGNPs) against stress-induced mice.

In the present study, our aim to investigate the effect of HPE and HPGNPS adult male mice on restraint-induced male mice relation to behavior and biochemical studies.

## MATERIALS AND METHODS

### Animals

Male Swiss albino mice (*Mus musculus*), weighing 20-30 g, were procured from the central animal house, Department of Experimental Medicine, Rajah Muthiah Medical College, Annamalai University, Annamalainagar. The animals were maintained at the central animal house and were fed on a standard balanced diet (Hindustan Lever, Bangalore, India) and provided with water ad libitum. All studies were conducted in accordance with the National Institute of Health Guide.

### Chemicals

Material used for the synthesis of gold nanoparticles are chloroauric acid (HAuCl_4_, Loba Chemicals Loba chemie pvt Ltd, Mumbai, India), HP leaves used in this study were received from Western Ghats of Nilgiris, Tamilnadu. Deionised double distilled water was used in this study.

### Plant material and preparation of extracts

*H. perforatum* plants were collected from Western Ghats of Nilgiris, Tamil Nadu and were botanically authenticated by the Department of Botany (Voucher Specimen Ac No. 2456), Annamalai University, Annamalainagar, Tamil Nadu. The leaves were air-dried at room temperature, finely powdered with auto-mix blender and stored in a deep freezer until the time of use. The methanolic extract was prepared using Soxhlet apparatus.

### Biosynthesis of gold nanoparticles:

One millimolar solution of 90 ml chloroauric acid at concentration of 10^-3^ M was prepared by dissolving DDW, kept in a 250 mL Erlenmeyer flask. Ten milliliters of HP (100 mg) supernatant was added to the chlorauric acid solution. The 95% of the bioreduction of AuCl_4_ – ions occurred within 30 min. The yellow-colored solution which it turned purple red slowly, indicating the formation of gold nanoparticles.

### Experimental design

The animals were divided into six groups, each consisting of six mice. Group I served as control, Group II received 200 mg/kg HPE (p.o.), Group III received 20 mg/Kg of HPGNPs Group IV served as restraint stress Group V 200 mg/kg HPE restraint and Group VI 20 mg/kg of HPGNPs restraint, respectively. HPE, HPGNPs (200 and 20 mg/kg, i.p.) were administered for 21 days and animals were subjected to restraint stress on 21 days. The doses were selected based on dose-dependent study.

### Restraint stress

Animals were restrained for 6-hr by taping all the four limbs on a board after putting them on their backs using zinc oxide hospital tape. Release was affected by unraveling the tape after moistening with acetone in order to minimize pain or discomfort. In unstressed group, the mice were kept in animal cage with soft bedding in the experimental room.[19]

### Behavioral assessments

#### T-Maze test

Spatial learning was recorded by T-maze on 21^st^ day of experimental period. The T-maze has a start arm and left and right arms and all arms are painted black inside. At the end of the two arms, a depression having a depth of 0.5 cm and the diameter of the depression is 5 cm. A food cup was placed in the depression situated to the right arm of the animal. The T-maze was located in a dimly illuminated room with a weak light (25 W). The animals were trained with the maze, food and food containers on two consecutive days before the commencement of the experiments held over a period of again two consecutive days. Three trials per animals were carried out. The animals were then deprived of food for 24 hours to make the animals for food motivation. During the experimental session, the number of errors and average time taken for each trial were noted. On the second day, the number of trials runs to reach the criterion of nine correct responses, and the number of errors and the average time taken for each trial run were noted.[20]

### Measurement of anxiety: mirror chamber test

The mirror chamber consisted of a wooden chamber having a mirror cube enclosed within it. The container box was (40 × 40 × 30.5) cm. Animal was placed at the distal corner of the mirror chamber at the beginning of the test. During the 5-min test session, following parameters were noted- a) latency to enter the mirror chamber and b) average time spent per entry in mirror chamber. An anxiogenic response was defined as decreased the number of entries and time spent in the mirror chamber.[21]

### Biochemical analysis

#### Estimation of lipid peroxidation

The quantitative measurement of lipid peroxidation in the whole brain was assessed as per method of Wills.[22]

### Assay of superoxide dismutase

SOD was assessed by the inhibition of formation of NADH- phenazine methosulphate nitroblue tetrazolium formazon.[23] The reaction was initiated by the addition of NADH after incubation for 90 s and stopped by the addition of glacial acetic acid. The color formed at the end of the reaction was extracted into the butanol layer and measured at 520 nm.

### Assay of catalase

CAT was assayed colorimetrically as per the method of Sinha.[24] Dichromate in acetic acid was converted to perchromic acid and then to chromic acetate when heated in the presence of H_2_O_2_. The chromic acetate formed was measured at 620 nm. The catalase preparation was allowed to split H_2_O_2_ for different periods of time. The reaction was stopped at different time intervals by the addition of a dichromate-acetic acid mixture and the remaining H_2_O_2_ was determined colorimetrically as chromic acetate.

### Assay of glutathione peroxidase

GPx was estimated as described by Rotruck *et al*.[25] A known amount of brain homogenate was allowed to react with H_2_O_2_ in the presence of GSH for a specified time period, then the remaining GSH was allowed to react with DTNB and the developed yellow color was measured at 412 nm.

### Estimation of reduced glutathione

GSH in brain homogenate was measured according to the method of Ellman.[26] This method is based on the development of a yellow color when 5,5’- dithio-bis-2-nitrobenzoic acid (DTNB) is added to compounds containing sulfhydryl groups.

### Statistical analysis

One way analysis of variance followed by Dunnet was employed for the analysis of behavioural variances and Biochemical parameters. *P*<0.05 was consideredsignificant.

## RESULTS

### Behavioral measurements

Table 1 shows the T-maze test and mirror chamber test. The control animals showed the normal spatial memory and anxiety behavior effect. The restraint-stressed animal showed significantly reduction in spatial memory and anxiety behaviors, as compared to unstressed group (*P*<0.05). Twenty-one days HPE and HPGNPs (200 and 20 mg/kg) treatment significantly improved spatial memory and anti-anxiety-like behavior as compared to control animals. HPGNPs-treated group significantly improved the activities as compared to HPE-treated group (*P*<0.05).

**Table 1:** Effect of *Hypericum perforatum* and HPGNP on behavior changes in stressed and unstressed animals

Groups	T-maze test (time taken in sec)	Mirror chamber test (Average time taken in sec)
Control	2.97 ± 0.75	50.53 ± 1.75
HPE	3.84 ± 0.42^a^	47.82 ± 2.42^a^
HPGNP	3.82 ± 0.31^a^	45.32 ± 0.71^a^
Restraint stress	41 ± 0.64^a^	10.16 ± 0.64^a^
(RS)		
HPE + RS	24.52 ± 0.45^b^	27.43 ± 0.45^b^
HPGNP+ RS	12.21. ± 1.25^b^^c^	39.21. ± 1.65^b^^c^

Values are expressed as mean ± SD for eight animals in each group; Values not sharing a common superscript differ significantly at *P*<0.05

a compared to control

b compared to control restraint

c ccompared to H. perforatum; HPE = H. perforatum extracts; HPGNP = H. perforatum gold nanoparticles, RS = restraint stress

Table 2 shows the TBARS and antioxidant status in the control and experimental groups. TBARS was significantly (*P*<0.05) increased in stress-induced group compared to the control group. HPE and HPGNPs treatment group, significantly (*P*<0.05) decrease the TBARS level compared to control group. HPGNPs-treated group significantly decrease TBARS level as compared to HPE-treated group (*P*<0.05). SOD, CAT, GPx and GSH activities were significantly decreased (*P*<0.05) in stress-induced animals as compared to the control group. These activities were significantly restored (*P*<0.05) in HPE and HPGNPs treatment group. HPGNPs-treated group significantly restored the activities as compared to HPE-treated group (*P*<0.05).

**Table 2:** Effect of *Hypericum perforatum* and HPGNP on biochemical changes in stressed and unstressed animals

Groups	TBARS (n moles/ mg of protein	SOD (μ/mg of protein)	CAT (μ/mg of protein)	Gpx (μ/mg of protein)	GSH (μg/100 g t issue)
Control	3.53 ± 0.25	3.94 ± 0.26	0.53 ± 0.35	13.84 ± 0.75	0.65 ± 0.05
HPE	3.57 ± 0.73^a^	3.68 ± 0.49^a^	0.59 ± 0.58^a^	13.92 ± 0.45^a^	0.57 ± 0.03^a^
HPGNP	3.67 ± 0.92^a^	3.84 ± 0.41^a^	0.57 ± 0.26^a^	13.79 ± 0.31^a^	0.62 ± 0.05^a^
RS	7.13 ± 0.63^a^	1.79 ± 0.57^a^	0.29 ± 0.17^a^	9.61 ± 0.03^a^	0.30 ± 0.07^a^
HPE + RS	5.47 ± 0.71^b^	2.48 ± 0.39^b^	0.40 ± 0.42^b^	10.72 ± 0.06^b^	0.41 ± 0.02^b^
HPGNP + RS	4.10 ± 0.28^b^^c^	3.72 ± 0.32^b^^c^	0.48 ± 0.31^b^^c^	12.32 ± 0.07^b^^c^	0.53 ± 0.01^b^^c^

Values are expressed as mean ± SD for eight animals in each group; Values not sharing a common superscript differ significantly at *P*<0.05

a compared to control

b compared to control restraint

c compared to H. perforatum; HPE = H. perforatum extracts; HPGNP = H. perforatum gold nanoparticles, RS = restraint stress

## DISCUSSION

In the present study, 6-hr restraint-stressed animal showed significant loss of memory and anxiety behavior, impaired memory activity indicating stress-induced neurobehavioral alterations.[27] Extracts of the medicinal plant HPE has been widely used in the therapy of various neurochemical disorders and have been reported to antidepressant, anxiolytic and cognitive effect.[28] In the present study the anxiety was assessed by mirror chamber test. After the administration of HPE and HPGNPs the improvement was better.[29] Khalifa *et al*. reported that significant improvement of the recognition and recall memory in rats treated with the crude preparation of HPE, the nootropic action,[30] was qualitatively comparable with that produced by piracetam.[31] and improved of cognitive functions,[32] learning and spatial memory[33] by the plant extract have also been reported. El –Sherbiny *et al*.[34] reported that HPE could be a novel type of antidepressant with memory-enhancing properties. So it is evident from this study as well as previous reports that HPE extract has definite neuroprotective action. Present study suggests its therapeutic potential against these stress-related altered behavioral states.

The study also reveals the existence of quantitative behavioral responses in 21 days stress-induced animals for the administration of HPE and HPGNPs. Stress-induced mice, subjected to the T-maze test and mirror chamber test, revealed a significant loss of spatial working memory and anxiety behavior. HPE and HPGNPstreatment for 21 days significantly improved behavior alterations, suggesting its neuroprotective effect against stressful conditions.

In the present study, stress-treated groups increase level of lipid per oxidation is indicated that the stress caused significant oxidative damage and depletes SOD, catalase, Gpx. and GSH activity. Tsuboi *et al*. reported an increased oxidative damage and weak antioxidant defense events are implicated in major depression.[35] Silva *et al*.[36] have reported that ethanolic extract of HPE contains many phenolic compounds namely flavonoids and phenolic acid suggesting that could have potent antioxidant property. Super oxide dismutase, catalase, glutathione peroxidase, reduced glutathione levels were significantly restored to significant levels. It suggests the possible role of HPE as an antioxidant and has been reported earlier by Hunt *et al*.[37] about the antioxidant effect of HPE against superoxide anion. They observed a free radical scavenging effect for most of the concentrations tested and postulated that this effect could not relate to its content in hypericin among other compounds. Flavonids, specifically quearcetin and its glycoside derivatives, are a major class of compounds present in the total ethanolic extract. These types of compounds are well-known antioxidants.[36] Sloley and Urichuk[38] reported that different standardized extracts of HPE demonstrated a free radical scavenging activity. Such free radical scavenging capacity was found to correlate with the content of several flavanodis including quercetin and hyperoside.[38] In the present study HPE and HPGNPs improved the antioxidant status in stressed mice.

In the present study, HPE and HPGNPs were effectively improved the antioxidant enzyme activities such as SOD, CAT, GPx and GSH in stress-treated animals. So, it is concluded that both the HPE and HPGNPs can exert a significant behavior and neuroprotective effect. But, the HPGNPs has a significant behavior and neuroprotective effect than compared to the HPE.

## CONCLUSION

The present study proved the neuroprotective activity of HPGNPs and HPEs against acute restrain stress causes neurobehavioral alterations and oxidative damage.

HPGNPs-treated group had modest activity when compared to HPE-treated group.
